# An induced pluripotent stem cell line (TRNDi009-C) from a Niemann-Pick disease type A patient carrying a heterozygous p.L302P (c.905 T > C) mutation in the *SMPD1* gene

**DOI:** 10.1016/j.scr.2019.101461

**Published:** 2019-05-15

**Authors:** Amanda Baskfield, Rong Li, Jeanette Beers, Jizhong Zou, Chengyu Liu, Wei Zheng

**Affiliations:** a National Center for Advancing Translational Sciences, National Institutes of Health, Bethesda, MD, USA; b iPSC core, National Heart, Lung and Blood Institute, National Institutes of Health, Bethesda, MD, USA; c Transgenic Core, National Heart, Lung and Blood Institute, National Institutes of Health, Bethesda, MD, USA

## Abstract

Niemann-Pick disease type A (NPA) is a rare autosomal recessive lysosomal storage disease caused by mutations in the *SMPD1* gene, which encodes for the protein acid sphingomyelinase. A human induced pluripotent stem cell (iPSC) line was generated from dermal fibroblasts of a 21-fetal-week-old female patient with NPA that has a heterozygous mutation of a p.L302P variant (c.905 T > C) using non-integrating Sendai virus technique. This iPSC line offers a useful resource to study the disease pathophysiology and as a cell-based model for drug development to treat NPA.

## Resource table.

**Table:** 

Unique stem cell line identifier	TRNDi009-C
Alternative name(s) of stem cell line	HT220C
Institution	National Institutes of Health National Center for Advancing Translational Sciences Bethesda, Maryland, USA
Contact information of distributor	Dr. Wei Zheng; Wei.Zheng@nih.gov
Type of cell line	iPSC
Origin	Human
Additional origin info	Age: 21-fetal-week-old Sex: Female Ethnicity: Caucasian
Cell Source	Fibroblasts
Clonality	Clonal
Method of reprogramming	Integration-free Sendai viral vectors
Genetic Modification	NO
Type of Modification	N/A
Associated disease	Niemann-Pick disease Type A (NPA)
Gene/locus	Gene: *SMPD1* Locus: 11p15.4 Mutation: p. L302P (c.905 T > C)
Method of modification	N/A
Name of transgene or resistance	N/A
Inducible/constitutive system	N/A
Date archived/stock date	2018
Cell line repository/bank	N/A
Ethical approval	NIGMS Informed Consent Form was obtained from patient at time of sample submission. Confidentiality Certificate: CC-GM-15–004

## Resource utility

This human induced pluripotent stem cell (iPSC) line is a useful tool for studies of disease phenotype and pathophysiology, and use as a cell-based disease model for drug development to treat patients with NPA.

## Resource details

Niemann-Pick disease type A (NPA) is a rare autosomal recessive, neuropathic lysosomal storage disease that is caused by mutations in the *SMPD1* gene, leading to a deficiency in acid sphingomyelinase (< 10% enzyme activity) in the cells (Schuchman and Wasserstein, 2015). This deficiency causes an accumulation of sphingomyelin in the lysosome, resulting in hepatosplenomegaly, developmental delay, interstitial lung disease, and cherry-red maculae (Schuchman and Wasserstein, 2015; Vanier, 2013). What differentiates NPA from Niemann-Pick disease type B (NPB) is the decrease in neurological function, such as the loss of acquired motor skills and a decrease in interest in their surrounding. These neurological symptoms are not commonly seen in NPB, which makes NPA the more severe form of the disease (Vanier, 2013). So far, there are no effective treatments or cures for NPA.

**Fig. 1.**
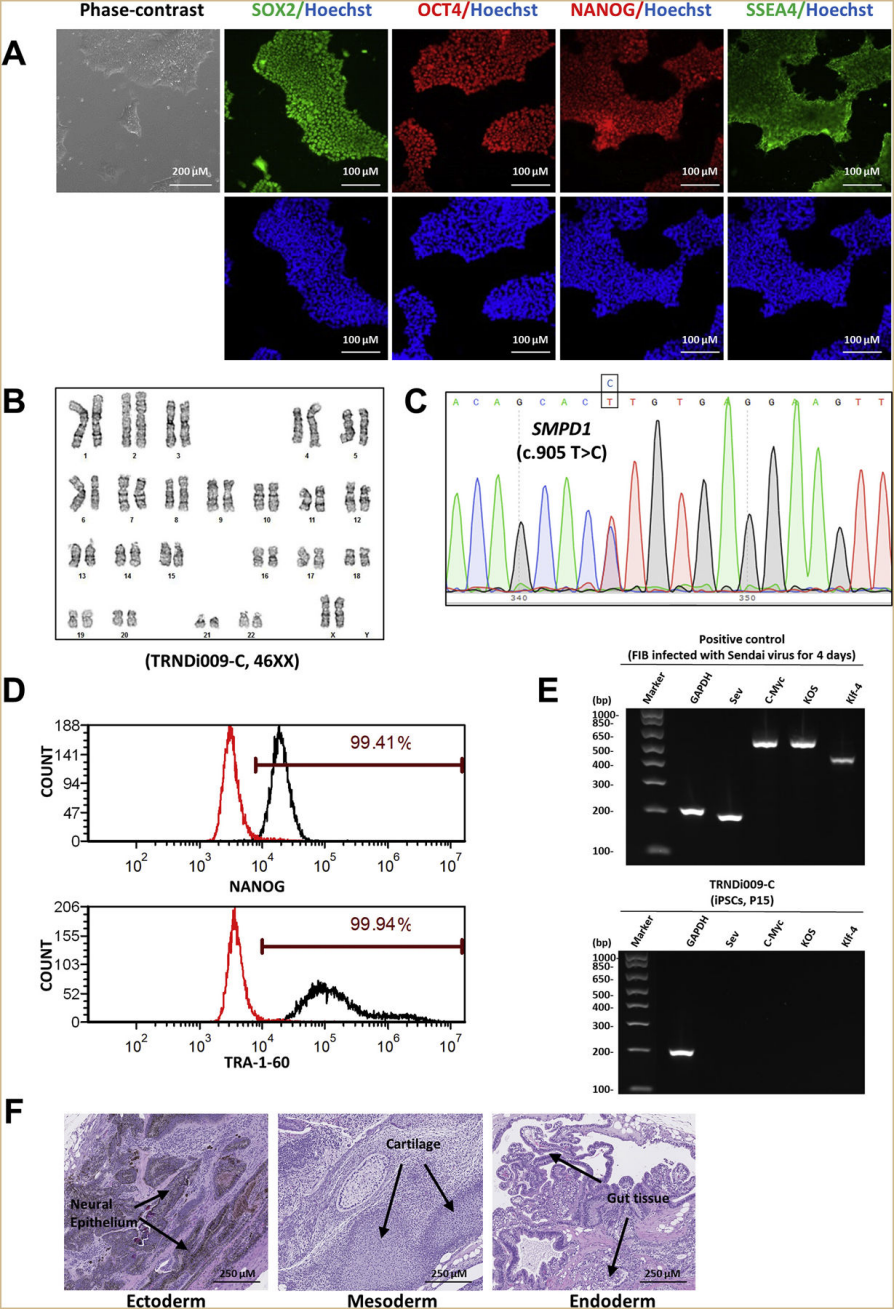
Characterization of TRNDi009-C iPSC line. A) Left: phase contrast imaging of TRNDi009-C colonies grown on Geltrex at passage 6. Right: Representative immunofluorescent images of iPSCs positive for stem cell markers: SOX2, OCT4, NANOG, and SSEA4. Nucleus is labelled with Hoechst 33342 (blue). B) Cytogenetic analysis showing a normal karyotype (46, XX). C) Detection of heterozygous mutation of a p.L302P (c.905 T > C) in exon 2 of the SMPD1 gene. D) Flow cytometry analysis of pluripotency protein markers: NANOG and TRA-1–60. E) RT-PCR verification for the clearance of the Sendai virus from reprogrammed cells. Sendai virus vector transduced fibroblasts were used as a positive control. F) Pathological analysis of teratoma from TRNDi009-C iPSC, showing a normal ectodermal, mesodermal, and endodermal differentiation.

In this study, a human iPSC line was established from skin dermal fibroblasts of a 21-fetal-week-old female patient (GM03252, Coriell Institute) (Table 1). To generate the iPSC line, the integration-free CytoTune-Sendai viral vector kit containing OCT ¾, KLF4, SOX2, and c-MYC pluripotency transcription factors was used to transduce the patient fibroblasts using methods previously stated (Chen et al., 2011; Beers et al., 2015). The iPSC line named TRNDi009-C was generated and the mutation of p.L302P (c.905 T > C) in exon 2 described by the Coriell Cell Repositories was confirmed in the TRNDi009-C iPSC line by Sanger sequencing of the PCR products harboring the single nucleotide variation (SNV) (Fig. 1C). The patient iPSCs exhibit a classical embryonic stem cell morphology (Fig. 1A) and carry normal karyotype (46, XX), as confirmed by G-banding karyotype analysis (Fig. 1B). Immunohistochemistry staining and flow cytometry analysis demonstrated high expression level of major pluripotency protein markers of NANOG, SOX2, OCT4, SSEA4, and TRA-1–60 (Fig. 1A and D). The Sendai virus (SeV) clearance was determined with reverse transcription polymerase chain reaction (RT-PCR) using SeV-specific primers and the vectors disappeared by passage 15 (Fig. 1E). Mycoplasma status was confirmed to be negative (Supplementary Fig. S1) and the cell line was authenticated using STR DNA profiling analysis, which demonstrated matching genotype at all 16 loci examined (information available with the authors). Furthermore, the pluripotency of this iPSC line was confirmed by teratoma formation experiment, which exhibited the ability to differentiate into cells/tissues of all three germ layers (ectoderm, neural epithelium; mesoderm, cartilage; endoderm, gut tissue) *in vivo* (Fig. 1F).

## Materials and methods

### Cell culture

A patient fibroblast line (GM03252) was obtained from Coriell Cell Repositories and cultured in DMEM (Thermo Fisher Scientific), supplemented with 10% fetal bovine serum (FBS), 100 units/mL penicillin, and 100 μg/mL streptomycin in a humidified incubator with 5% CO_2_ at 37 °C. TRNDi009-C iPSCs were cultured in mTeSR1 medium (StemCell Technologies) on Geltrex (Thermo Fisher Scientific)-coated plates. The cells were maintained at 5% CO_2_, 5% O_2_ at 37 °C and were passaged with 0.5 mM ethylenediaminetetraacetic acid (EDTA) when colonies were approximately 70% confluent.

### Reprogramming of human skin fibroblasts

Fibroblast cells were reprogrammed into iPSCs using the integration-free CytoTune Sendai viral vector kit (A16517, Thermo Fisher Scientific) following the method previously stated (Chen et al., 2011; Beers et al., 2015).

### Immunocytochemistry staining

For immunofluorescence staining, iPSCs cultured in 96-well plate were fixed with 4% paraformaldehyde for 30 mins at RT, permeabilized with 0.3% Triton X-100 in Dulbecco’s phosphate-buffered saline (DPBS) for 15 mins, and washed with DPBS. Cells were blocked using Image-iT™ FX signal enhancer (Thermo Fisher Scientific) for 30 mins at room temperature and incubated with primary antibodies, including SOX2, OCT4, NANOG and SSEA4, overnight at 4 °C. Cells were then washed and incubated with corresponding secondary antibody conjugated with Alexa Fluor 488 or Alexa Fluor 594 for 1 h at room temperature (antibodies used are listed in Table 2). Cells were washed and stained with Hoechst 33342 for 15 mins and imaged using an INCell Analyzer 2200 imaging system (GE Healthcare) with 20× objective lens and Texas Red, FITC and DAPI filter sets.

### Flow cytometry analysis

The iPSCs were harvested using TrypLE Express enzyme (Thermo Fisher Scientific). Cells were fixed with 4% paraformaldehyde for 10 mins at room temperature and then washed with DPBS. Before fluorescence-activated cell sorting analysis, cells were permeabilized with 0.2% Tween-20 in DPBS for 10 mins at room temperature and stained with fluorophore-conjugated antibodies for 1 h at 4 °C on a shaker. Relative fluorophore-conjugated animal nonimmune Immunoglobulin was used as the negative control. Antibodies and nonimmune immunoglobulins used are listed in Table 2). Cells were then analyzed on a BD Accuri™ C6 Flow Cytometry system (BD Biosciences).

### G-banding karyotype

The G-banding karyotype analysis was performed at WiCell Research Institute (Madison, WI, USA). Cell harvest, slide preparation, and G-banding karyotype were performed using standard cytogenetic protocols. Cells were incubated with ethidium bromide and colcemid and placed in a hypotonic solution, followed by fixation. Metaphase cell preparations were stained with Leishman’s stain. A total of 20 randomly selected metaphases were analyzed by G-banding.

### Gene analysis of SMPD1

The gene analysis of *SMPD1* was conducted through Applied StemCell (Milpitas, CA, USA). Genomic DNA was extracted from iPSC line TRNDi009-C followed by PCR amplification using MyTaqTM Red Mix (Bioline, Taunton, MA). Gene Amplifications were carried out using the following program: 95 °C, 2 mins; 35 cycles of [95 °C, 15 s; 60 °C, 15 s; 72 °C, elongation duration varies by amplificon size], 72 °C, 5 mins; 4 °C, indefinite. PCR product was subsequently sequenced by Sanger sequencing analysis to identify potential imutations. The specific primers for gene amplification and sequencing are listed in Table 2.

### Testing for Sendai reprogramming vector clearance

Total RNA was isolated from iPSCs TRNDi009-C of passage 15 using RNeasy Plus Mini Kit (Qiagen). Human fibroblasts (Coriell Institute, GM05659) after transduction with Sendai virus for 4 days were used as positive control. A total of 1 μg RNA/reaction was reverse transcribed with SuperScript™ III First-Strand Synthesis SuperMix kit, and PCR was performed using Platinum II Hot Start PCR Master Mix (Thermo Fisher Scientific) with the primers listed in Table 2. The products were then loaded into an *E*-Gel® 1.2% with SYBR Safe™ gel and run at 120 V electric field. Finally the image was collected using G: Box Chemi-XX6 gel doc system from Syngene (Frederick, MD).

### Mycoplasma detection

Mycoplasma testing was performed and analyzed using the Lonza MycoAlert kit, following the protocol from the company (ratio B/A > 1.2, mycoplasma positive; 0.9–1.2, results ambiguous; < 0.9, mycoplasma negative).

### Short tandem repeat (STR) DNA profile analysis

Patient fibroblasts and derived iPSC lines were sent to the WiCell Institute for STR analysis. Briefly, the Promega PowerPlex® 16 HS System (Promega, Madison, WI) was used in multiplex polymerase chain reaction (PCR) to amplify fifteen STR loci (D5S818, D13S317, D7S820, D16S539, vWA, TH01, TPOX, CSF1PO, D18S51, D21S11, D3S1358, D8S1179, FGA, Penta D, Penta E) plus a gender determining marker, Amelogenin (AMEL). The PCR product was capillary electrophoresed on an ABI 3500xL Genetic Analyzer (Applied Biosystems) using the Internal Lane Standard 600 (ILS 600) (Promega, Madison, WI). Data were analyzed using GeneMapper® v 4.1 software (Applied Biosystems).

### Teratoma formation assay

Human iPSCs cultured in 6-well plates were dissociated with DPBS containing 0.5 mM EDTA and approximately 1 × 10^7^ dissociated cells were collected and resuspended in 400 μl culture medium supplied with 25 mM HEPES (pH 7.4) and stored on ice. Then, 50% volume (200 μl) of cold Matrigel (354277, Corning) was added and mixed with the cells. The mixture was injected subcutaneously into NSG mice (JAX No. 005557) at 150 μl per injection site. Visible tumors were removed 6–8 weeks post injection, and were immediately fixed in 10% Neutral Buffered Formalin. The fixed tumors were embedded in paraffin and stained with hematoxylin and eosin. Images were collected using the NanoZoomer Digital Pathology software (Hamamatsu).

Supplementary data to this article can be found online at https://doi.org/10.1016/j.scr.2019.101461.

## Supplementary Material

1

## Figures and Tables

**Table 1:** Characterization and validation.

Classification	Test	Result	Data
Morphology	Photography	Normal	Fig. 1 Panel A
Phenotype	Immunocytochemistry	SOX2, OCT4, NANOG, SSEA-4	Fig. 1 Panel A
	Flow cytometry	TRA-1–60 (99.94%); NANOG (99.41%)	Fig. 1 Panel D
Genotype	Karyotype (G-banding) and resolution	46XX, Resolution: 425–500	Fig. 1 Panel B
Identity	Microsatellite PCR (mPCR) OR	Not performed	N/A
	STR analysis	16 sites tested, all sites matched	Available from the authors
Mutation analysis	Sequencing	*SMPD1*, p.L302P (c.905 T > C)	Fig. 1 Panel C
	Southern Blot OR WGS	N/A	N/A
Microbiology and virology	Mycoplasma	Mycoplasma testing by luminescence. Negative	Supplementary Fig. S1
Differentiation potential	Teratoma formation	Teratoma with three germlayers formation. Ectoderm (neural epithelium); Mesoderm (cartilage); Endoderm (gut tissue)	Fig. 1 Panel F
Donor screening	HIV 1 + 2 Hepatitis B, Hepatitis C	N/A	N/A
Genotype additional info	Blood group genotyping	N/A	N/A
	HLA tissue typing	N/A	N/A

**Table 2:** Reagents details.

Antibodies used for immunocytochemistry/flow-cytometry			
	Antibody	Dilution	Company Cat # and RRID
*Pluripotency Markers*	*Mouse anti-SOX2*	*1:50*	* R&D systems, Cat# MAB2018, RRID:AB_358009*
*Pluripotency Markers*	*Rabbit anti-NANOG*	*1:400*	* Cell Signaling Technology, Cat# 4903, RRID:AB_10559205*
*Pluripotency Markers*	*Rabbit anti-OCT4*	*1:400*	* Thermo Fisher Scientific, Cat# A13998, RRID:AB_2534182*
*Pluripotency Markers*	*Mouse anti-SSEA4*	*1:1000*	* Cell Signaling Technology, Cat# 4755, RRID:AB_1264259*
*Secondary antibodies*	*Donkey anti-Mouse IgG (Alexa Fluor 488)*	*1:400*	* Thermo Fisher Scientific, Cat# A21202, RRID:AB_141607*
*Secondary antibodies*	*Donkey anti-Rabbit IgG (Alexa Fluor 594)*	*1:400*	* Thermo Fisher Scientific, Cat# A21207, RRID:AB_141637*
*Flow cytometry antibodies*	*Anti-Tra-1–60-DyLight 488*	*1:50*	* Thermo Fisher Scientific, Cat# MA1–023-D488X, RRID:AB_2536700*
*Flow cytometry antibodies*	*Anti-Nanog-Alexa Fluor 488*	*1:50*	* Millipore, Cat# FCABS352A4, RRID:AB_10807973*
*Flow cytometry antibodies*	*Mouse-IgM-DyLight 488*	*1:50*	* Thermo Fisher Scientific, Cat# MA1–194-D488, RRID:AB_2536969*
*Flow cytometry antibodies*	*Rabbit IgG-Alexa Fluor 488*	*1:50*	* Cell Signaling Technology, Cat# 4340S, RRID:AB_10694568*
*Flow cytometry antibodies*	*Mouse IgG3-FITC*	*1:50*	* Thermo Fisher Scientific, Cat# 11–4742-42, RRID:AB_2043894*

Primers			
	Target		Forward/Reverse primer (5′-3′)

*Sev specific primers (RT-PCR)*	* Sev/181 bp*		*GGATCACTAGGTGATATCGAGC/ACCAGACAAGAGTTTAAGAGATATGTATC*
*Sev specific primers (RT-PCR)*	* KOS/528 bp*		*ATGCACCGCTACGACGTGAGCGC/ACCTTGACAATCCTGATGTGG*
*Sev specific primers (RT-PCR)*	* Klf4/410 bp*		*TTCCTGCATGCCAGAGGAGCCC/AATGTATCGAAGGTGCTCAA*
*Sev specific primers (RT-PCR)*	* C-Myc/523 bp*		*TAACTGACTAGCAGGCTTGTCG/TCCACATACAGTCCTGGATGATGATG*
*House-Keeping gene (RT-PCR)*	* GAPDH/197 bp*		*GGAGCGAGATCCCTCCAAAAT/GGCTGTTGTCATACTTCTCATGG*
*Targeted mutation analysis (PCR)*	* SMPD¼54 bp*		*CTGAAGGTGAGCACTGAAGG/TGGTGAGAAATCAGAGGCAG*

## References

[R1] Beers J, Linask KL, Chen JA, Siniscalchi LI, Lin Y, Zheng W, Rao M, Chen G, 2015. A cost-effective and efficient reprogramming platform for large-scale production of integration-free human induced pluripotent stem cells in chemically defined culture. Sci. Rep 5, 11319. 26066579 10.1038/srep11319 PMC4464084

[R2] Chen G, Gulbranson DR, Hou Z, Bolin JM, Ruotti V, Probasco MD, Smuga-Otto K, Howden SE, Diol NR, Propson NE, Wagner R, Lee GO, Antosiewicz-Bourget J, Teng JM, Thomson JA, 2011. Chemically defined conditions for human iPSC derivation and culture. Nat. Methods 8, 424–429. 21478862 10.1038/nmeth.1593 PMC3084903

[R3] Schuchman EH, Wasserstein MP, 2015. Acid sphingomyelinase deficiency. In: GeneReviews National Library of Medicine, National Institutes of Health, NCBI Bookshelf, pp. 16.

[R4] Vanier MT, 2013. Niemann-Pick diseases. In: Handbook of Clinical Neurology vol. 113. pp. 5. 10.1016/B978-0-444-59565-2.00041-1 23622394

