# Supplementary figures and images for: An induced pluripotent stem cell line (TRNDi009-C) from a Niemann-Pick disease type A patient carrying a heterozygous p.L302P (c.905 T > C) mutation in the SMPD1 gene
Source: Stem Cell Res. Author manuscript; Available in PMC 2019 Aug 8. (PMC6686851; doi:10.1016/j.scr.2019.101461)

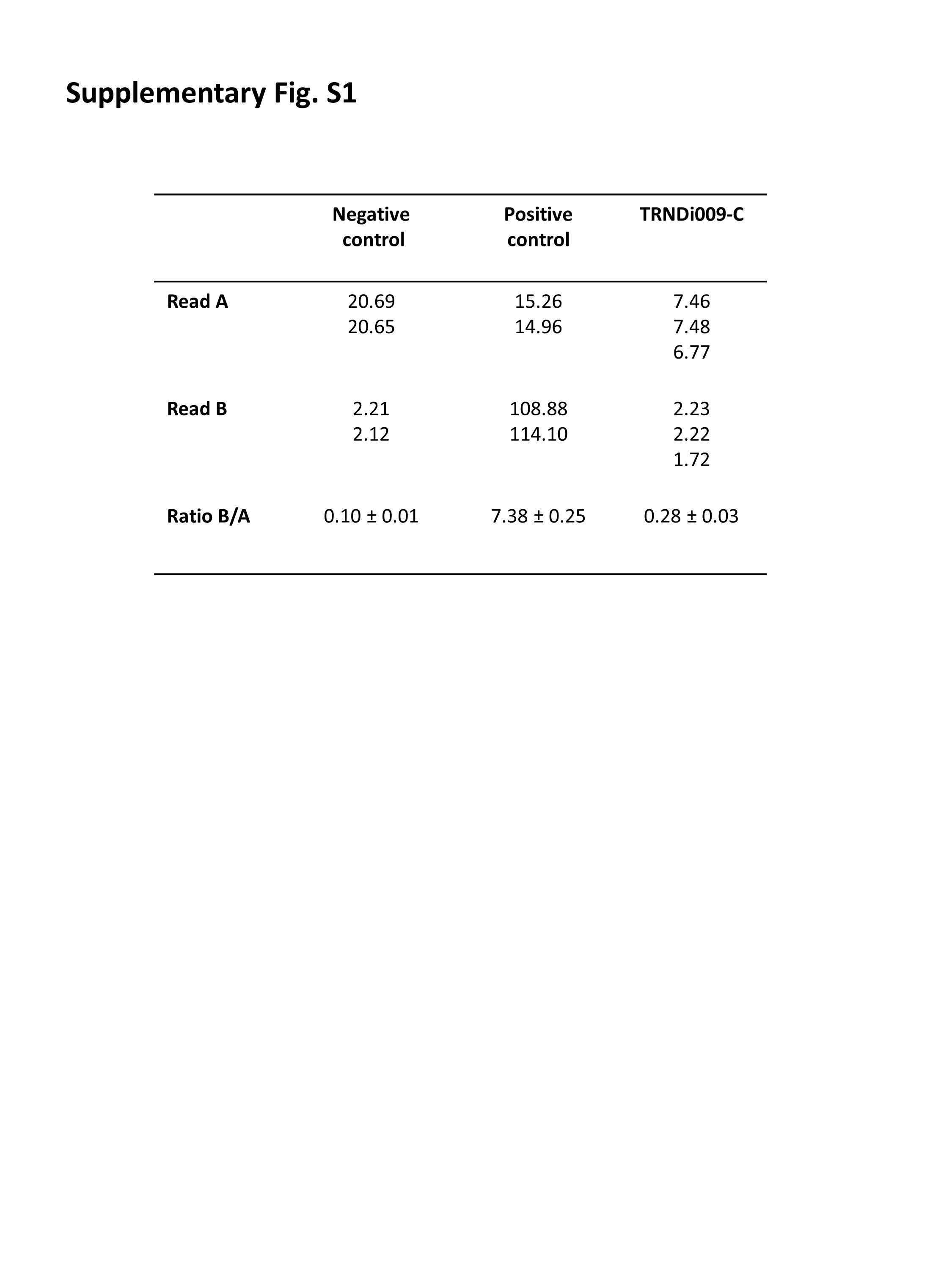

Supplement: 1 [file NIHMS1534105-supplement-1.tif]
